# Knockout analysis of *period* and *timeless* and EGFP-based visualization of *per*-expressing clock cells in the cricket circadian clock

**DOI:** 10.1186/s40851-026-00267-6

**Published:** 2026-07-07

**Authors:** Kenji Tomioka, Shintaro Inoue, Taro Mito, Yoshiyuki Moriyama, Taishi Yoshii

**Affiliations:** 1 Graduate School of Natural Science and Technology, Okayama University, Okayama, 700-8530 Japan; 2 Bio-Innovation Research Center, Tokushima University, Ishii, Tokushima, 779-3233 Japan; 3 Department of Natural Sciences, Kawasaki Medical School, Kurashiki, 701-0192 Japan; 4 Graduate School of Environmental, Life, Natural Science and Technology, Okayama University, Okayama, 700-8530 Japan

**Keywords:** Circadian clock, Cricket, Genome editing, Locomotor rhythm, *period*, *per*-less oscillation, *timeless*

## Abstract

In the present study, we generated crickets with knockout of either *period* (*per*) or *timeless* (*tim*) gene by CRISPR/Cas9-based genome editing. We also identified a naturally occurring *per*^-^ mutant lacking a large coding region including PAS domains. To examine possible synergistic effects, a *per*^-^ and *tim*^KO^ double mutant was generated by applying genome editing to the *per*^-^ crickets. Under constant darkness (DD), *tim*^KO^ crickets exhibited a locomotor rhythm with a free-running period of 23.06 ± 0.20 h (mean ± SD), which was significantly shorter than that of the parental strain (23.78 ± 0.12 h). By contrast, *per*^KO^ and *per*^-^ crickets showed basically similar phenotype of locomotor rhythm: they exhibited an arrhythmic pattern during the first two to three weeks after transfer to DD but subsequently showed a complex rhythmic pattern with one or multiple components with significantly longer free-running periods (33.35 ± 10.72 h). In the *per*^-^;*tim*^KO^ double mutants, approximately 60% of individuals became arrhythmic, while the remaining 40% exhibited complex rhythms with extremely longer free-running periods (37.0 ± 9.17 h) under DD. These results suggest the existence of an underlying oscillatory mechanism that is responsible for regulating locomotor rhythms independently of the canonical *per/tim* feedback loop. Furthermore, we generated a reporter line on a *per*^−^ background by knocking *egfp* into exon 1 of the *per* gene, allowing *egfp* expression to report *per* transcription. EGFP expression was detected in three distinct clusters of cells within the optic lobe: two located along the dorsal and ventral boundaries between the lamina and medulla neuropils, and one situated near the accessory medulla. These findings raise the possibility that these form part of the circadian clock network that governs circadian locomotor rhythms.

**Supplementary information:**

The online version contains supplementary material available at 10.1186/s40851-026-00267-6.

## Background

It is generally accepted that the circadian clock consists of transcriptional-translational feedback loops (TTFLs). In many insects, the main TTFL includes *per*, *tim*, *Clock* (*Clk*), and *cycle* (*cyc*). In some insects, including hymenopterans, however, *tim* is absent, and the TTFL instead relies on the mammalian-type *cryptochrome* (m-*cry* or *cry2*) as the principal negative regulator. *Clk* and *cyc* encode the transcriptional activators, CLK and CYC, respectively, which form a CLK/CYC heterodimer and activate transcription of *per* and *tim* by binding to E-boxes in their promoter regions during late day to early night [1, 2]. The resultant proteins PER and TIM accumulate during the night, heterodimerize to form a PER/TIM complex, which enters the nucleus and suppresses their own transcription through inactivation of CLK/CYC [1, 2]. However, there are several lines of evidence for the existence of an oscillatory mechanism that functions without *per* or *tim*. For example, the circadian cuticular deposition rhythm is maintained in *per*^0^ and *tim*^0^ mutant fruit flies *Drosophila melanogaster* [3]. *per*^0^ mutant *Drosophila* exhibits locomotor rhythms under LD within a circadian range [4]. Under temperature cycles, *per*^0^ and *tim*^0^ flies show a locomotor rhythm and a characteristic phase relationship to the temperature cycles [5]. However, the *per*/*tim*-less oscillatory mechanism still remains unresolved.

In the cricket, *Gryllus bimaculatus*, the clock mechanism has been hypothesized to consist of two oscillatory loops, namely the *per*/*tim*-loop and a *cry*-based loop. In the latter, variant proteins of CRY2 and/or CRY1 are thought to form a complex that may function as a negative element to suppress their own transcription through inactivation of CLK/CYC [6]. Several experimental facts suggest that the *per*/*tim*-loop and the *cry*-based loop likely function solely only when the other loop is dysfunctional [6, 7]. However, the hypothesis is based on the results of RNAi-mediated gene silencing and awaits testing of its validity by using gene knockout strains.

Another important issue to be addressed is the identification of the circadian clock cells in this cricket. We have shown that the optic lobe is the locus of the circadian clock by a series of behavioral and electrophysiological experiments. The removal of the optic lobe resulted in a loss of circadian locomotor rhythms [8], the circadian ERG rhythms persisted even after the optic lobe was surgically separated from the brain [9], and the optic lobe maintains its circadian neural activity in isolated and in vitro cultured conditions [10, 11]. However, the cells that are responsible for generating the circadian rhythm remain to be identified in the optic lobe.

In the present study, we generated knockouts of the *per* and *tim* genes using the CRISPR/Cas9-mediated genome editing technology in the cricket *G. bimaculatus*. With the knockout crickets, we examined the role of *per* and *tim* genes in the circadian rhythm generation and obtained evidence suggesting the existence of an oscillatory mechanism that functions without *per* and *tim*. We also generated a cricket strain that carries an *egfp* gene knocked into exon 1 of *per*. With this knock-in strain, we have identified the clock cells that express PER in the optic lobe.

## Materials and methods

### Animals

The white-eyed mutant strain of cricket *G. bimaculatus* was used as the wild-type background [12]. Only male crickets were used in the experiments and were reared under a 12 h light:12 h dark cycle (LD12:12) at a constant temperature of 25 °C or 29 °C. They were fed ad libitum with laboratory chow (Nihon Crea, CA-1) and water through dampened cotton.

### Genome information

The *G. bimaculatus* genome information referenced in this study was based on the assemblies of the white-eyed strain reported by Ylla et al. [13] and Kataoka et al. [14]. These genome sequences were used to identify the exon–intron structures, design guide RNAs, and design primers for *per* (AB375516) and *tim* (AB548625).

### Generation of *per* and *tim* frameshift mutant strains (*tim*^KO^ and *per*^KO^)

CRISPR/Cas9-mediated genome editing was applied to *tim* and *per*, following previously described protocols [15, 16]. A guide RNA (gRNA) specific to *per* exon 2 and *tim* exon 3 (Supplementary Table S1), both located within their coding sequences, was designed using the web tool CRISPOR [17]. A solution containing a ribonucleoprotein complex (RNP) composed of 0.74 μM guide RNA and 0.31 μM Cas9 protein (Alt-R S.p. HiFi Cas9 Nuclease V3; IDT), together with injection buffer (1.4 mM NaCl, 0.07 mM Na₂HPO₄, 0.03 mM KH₂PO₄, and 4 mM KCl), was microinjected into eggs of the wild-type strain within 3 h after oviposition. The eggs were kept on moist paper until hatching. The hatched G₀ (genome-edited generation) individuals were reared to adulthood and crossed with wild-type crickets. Genomic DNA was extracted from the legs of G₁ nymphs, and genomic PCR and sequencing of *per* exon 2 and *tim* exon 3 were performed using the primers listed in Supplementary Table S2. Heterozygous frameshift mutants carrying identical indel sequences were crossed to produce the G₂ generation, which contained homozygotes at the expected Mendelian ratio. The G₃ homozygous strain with a uniform genetic background was established by crossing these homozygous G₂ individuals.

### Identification of naturally occurring *per* mutant (*per*^-^) and generation of a *per*^-^;*tim*^KO^ double mutant strain

In the white-eye wild-type strain of *G. bimaculatus*, individuals harboring a naturally occurring incomplete *per* allele were identified by their lack of genomic PCR amplification with primer sets targeting exons 2 and 3 of *per* (Supplementary Table S2). These males and females were crossed to establish the *per*^-^ strain. For the analysis of *per* transcripts in the *per*^-^ strain, total RNA was extracted from adult heads using TRIzol (Invitrogen). The RNA was subjected to genomic DNA removal and cDNA synthesis using the SuperScript IV VILO Master Mix with ezDNase Enzyme (Invitrogen). RT-PCR of *per* was performed using the primer sets designed for exon 1 and exon 16 (Supplementary Table S2). The amplified products were TA-cloned into the pGEM-T Easy vector (Promega, USA) and subjected to sequencing.

By performing genome editing of *tim* exon 3, as described in the preceding section, with the *per*^-^ strain as the genetic background, the *per*^-^;*tim*^KO^ strain was obtained.

### Generation of a *per* enhancer-trap strain carrying a *per* mutation (*per*^*-/egfp*KI^)

Insertion of a reporter gene expression cassette containing a minimal promoter into a gene locus enables trapping of endogenous enhancer activity, thereby allowing visualization of gene expression [18–20]. To visualize *per* expression, an EGFP expression vector containing the endogenous cytoplasmic *actin* promoter of *G. bimaculatus* (pUC57; *DsRed*bait*-G’act-eGFP* [18]), was knocked into the first exon of *per* by non-homologous end joining (NHEJ)-based CRISPR/Cas9 genome editing, using the *per*^-^ strain as the genetic background. The guide RNA targeting *per* exon 1 (Supplementary Table S1) was designed using the web tool CRISPOR [17]. A guide RNA targeting the *DsRed* region of the vector (Supplementary Table S1) was also used to linearize the expression vector. Injection solutions containing RNPs (0.5 μM gRNA + 0.2 μM Cas9) against *per* and *DsRed*, and 67 ng/μL expression vector (final concentrations) were prepared and microinjected into eggs. Knock-in events were first confirmed by detecting EGFP fluorescence in the G₀ generation. G₀ adults exhibiting EGFP fluorescence were intercrossed to obtain the G₁ generation. Germline transmission of the expression cassette was evaluated based on EGFP fluorescence. G₁ heterozygotes displaying fluorescence were crossed with the *per*^-^ background strain to generate G₂ heterozygotes with a uniform cassette-insertion configuration. Intercrossing these G₂ individuals yielded G₃ homozygotes, thereby establishing the *per*^-*/egfp*KI^ strain. The knock-in configuration was verified by genomic PCR using primers *per*-exon1-Fw and *per*-intron1-Rv (Supplementary Table S2) that amplify the genomic regions flanking the insertion site, followed by sequencing.

### mRNA measurement

mRNA levels of *per*, *tim*, and *cry2* (LC202053) genes were measured using quantitative real-time RT-PCR (qPCR). Total RNA was extracted and purified from two optic lobes of adult males with TRIzol Reagent (Invitrogen, Carlsbad, CA, USA). To remove contaminating genomic DNA, the total RNA was treated with DNase I (Takara, Kusatsu, Japan). Approximately 500 ng of total RNA of each sample was reverse transcribed with random hexamers using PrimeScript RT reagent Kit (Takara). Real-time PCR was performed using the Mx3000P QPCR System (Agilent Technologies, Santa Clara, CA, USA) using THUNDERBIRD® Next SYBR^TM^ qPCR Mix (TOYOBO, Osaka, Japan), including SYBR Green with primers designed for *per*, *tim*, *cry2* and *rpl18a* (DC448653) (Supplementary Table S3). The primers for *tim* and *per* were designed outside the mutated regions in the mutant strains (*tim*^*KO*^, *per*^*KO*^, *per*^*−*^); therefore, transcripts of these genes can still be detected even when mutations are present. In all cases, a single expected amplicon was confirmed by melting curve analysis. The quantification was made based on a standard curve obtained with known amounts of templates. The results were analyzed using the software associated with the instrument. The values were then normalized to those of *Gb’rpl18a*, an internal reference gene, at each time point. Results of four independent experiments were used to calculate the mean ± SEM.

### Behavioral analysis

The locomotor activity of individual animals was recorded within a transparent plastic activity chamber (18 × 9 × 4.5 cm) with a rocking substratum. A magnetic reed switch sensed the rocking movement of the substratum caused by a moving cricket. The number of rocks was recorded every 6 min by a computerized system. Water and food were provided *ad libitum*. The activity chambers were placed in an incubator (MIR-153, Sanyo Biomedica, Osaka, Japan), in which temperature was kept at 25 ± 0.5 °C and the desired lighting regimen was provided by a cool white fluorescent lamp connected to an electric timer. The light intensity was 600 to 1000 lux at the animal’s level, varying with the proximity to the lamp. The raw data were displayed as conventional double-plotted actograms to judge activity patterns. The free-running periods under constant darkness (DD) were calculated by the χ^2^ periodogram [21] using actogramJ [22]. If consecutive two or more conservative points of a peak in the periodogram appeared above the 5% confidence level, the period of the peak was considered as statistically significant [23]. The power of the rhythm was quantified as the difference between the peak value and the 5% confidence threshold.

### Immunohistochemistry

The brains of first-instar nymphal *per*^-/*egfp*KI^ crickets were dissected in phosphate-buffered saline (PBS) and immediately fixed in 4% paraformaldehyde in PBS at room temperature (RT, around 25°C) for 1 h. The fixed brains were washed three times with PBS containing 0.5% Triton X-100 (PBS-T), then blocked in PBS-T containing 5% normal donkey serum for 1 h at RT, and incubated with primary antibodies at 4 °C for 48 h. After washing six times with PBS-T, the brains were incubated with secondary antibodies at 4 °C for 16 h and washed six more times in PBS-T. To render the cricket brains transparent for subsequent confocal imaging, a rapid tissue clearing protocol was employed using the RapiClear reagent (RapiClear 1.47, SunJin Lab Co., Taiwan). The brains were immersed in the RapiClear reagent at RT for 10 min and mounted in the same RapiClear reagent. Staining was visualized using a laser scanning confocal microscope (FV3000; Olympus, Tokyo, Japan). The primary antibodies used were as follows: chicken anti-GFP (1:1000; Rockland, Limerick, PA, USA), and rabbit anti-PDF (1:15000) [24]. Fluorescence-conjugated secondary antibodies were used at a concentration of 1:1000: Alexa Fluor®-488 (goat anti-chicken) and Alexa Fluor®-647 nm (goat anti-rabbit) (all from Life Technologies, Carlsbad, CA, USA).

### Statistics

Statistical analyses were conducted as follows: a t-test was used to compare the means of two groups; Dunnett’s test was applied for comparisons between multiple test groups and a single control group; one-way ANOVA was used to assess differences among more than two groups; and differences in proportions were assessed using the likelihood ratio G-test.

## Results

### Establishment of genome editing-mediated *per* and *tim* knockout and *egfp* knock-in strains

**Fig. 1:**
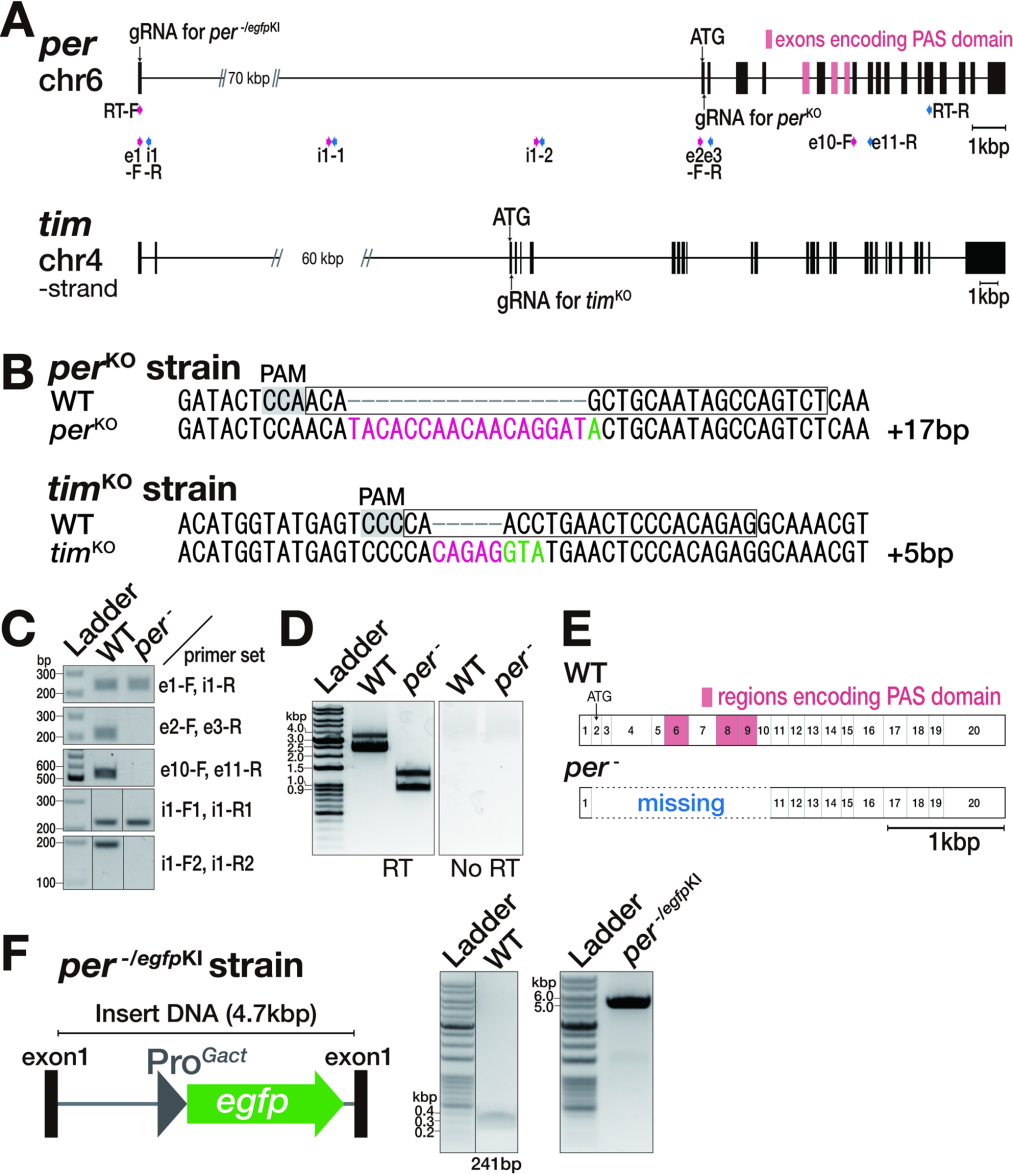
Gene structure of *per* and *tim* and the genetic characteristics of the strains used in this study. (**A**) Exon-intron structures of *per* and *tim*. In the *per*^-^ allele, red and blue arrows indicate the forward and reverse primers, respectively, used in subsequent experiments. (**B**) The *per*^KO^ and *tim*^KO^ strains generated by genome editing harbor frameshift mutations. (**C**) Genomic PCR analysis in the *per*^-^ strain. The primer names correspond to panel **A.** The letter e in the primer name denotes an exon, while i denotes an intron. (**D**) Two distinct *per* transcripts with differing lengths of the 5’ UTR (first exon) were detected in both wild-type and *per*^-^ mutant crickets. The *per*^-^ strain possesses shorter *per* transcripts than the wild-type. Sequence data for *per*^-^ are shown in Supplementary Figure S1. (**E**) Schematic representation of the structure of *per* transcripts. The *per* transcript derived from *per*^-^ lacks the sequences encoding the initiation methionine and the PAS domain, consistent with *per*^-^ representing a null strain. The panel shows the shorter transcript detected in *per*^-^ strain in **D.** The numbers in the panel indicate the corresponding exon numbers. (**F**) Schematic diagram of *egfp* expression cassette insertion patterns in the *per*^-/*egfp*KI^ strain. PCR and sequencing results (Supplementary Figure S2) confirm that the expression cassette is inserted as a single copy in the forward orientation

The *per* locus spans 96 kb on chromosome 6 and contains 20 exons, while the *tim* locus extends across 107 kb on chromosome 4 and comprises 27 exons (Fig. 1A). Translation of the PER and TIM proteins initiates from exon 2 of *per* and exon 3 of *tim*, respectively. To generate frameshift mutant strains of *per* and *tim*, we performed CRISPR/Cas9-mediated genome editing using gRNAs targeting regions downstream of the translation start codon in exon 2 of *per* and exon 3 of *tim* (Fig. 1A). Consequently, we obtained homozygous knockout strains, designated *per*^KO^ and *tim*^KO^, which carry a 17 bp insertion in exon 2 of *per* and a 5 bp insertion in exon 3 of *tim*, respectively (Fig. 1B). These insertions introduce frameshifts that abolish the function of the corresponding genes.

We found that within a laboratory-maintained wild-type population, some individuals carried a naturally occurring *per* mutant allele in which exon 2 and 3 of the *per* failed to amplify in the genome (Fig. 1C). We collected these individuals and established the strain, designated *per*^-^. In *per*^-^, the genomic region from the latter half of intron 1 through exon 11 failed to be amplified by PCR, whereas exon 1 was amplified (Fig. 1C). Two distinct transcripts with different lengths of the 5’UTR (first exon) were detected in both wild-type and *per*^-^ mutant crickets, similar to *Drosophila per*, which produces two alternative splicing variants in its 3’UTR [25, 26]. In the *per*^-^ strain, both amplified transcripts were markedly shorter than those in the wild-type (Fig. 1D) and lacked sequences corresponding to exons 2–10 (Supplementary Figure S1). As a result, they do not encode the initiation methionine and the PAS domains (Fig. 1E). These findings indicate that *per*^-^ is a null strain.

To visualize cells expressing *per*, we inserted a 4.7 kbp *egfp* expression cassette into exon 1 of the *per*^-^ allele via NHEJ–based CRISPR/Cas9 genome editing (Fig. 1F). Given that feedback regulation is expected to be lost in the *per*^*−*^ strain, the EGFP signal was expected to be more stable. In the wild-type genome, PCR amplification of the knock-in target region yielded a ~ 0.2 kbp fragment, whereas the *per*^*-/egfp*KI^ strain produced an amplicon of ~ 5 kbp. Together with the sequence analysis (Supplementary Figure S2), these results confirmed that the *egfp* expression cassette was inserted as a single copy in the forward orientation.

### Locomotor rhythm of control crickets

**Fig. 2:**
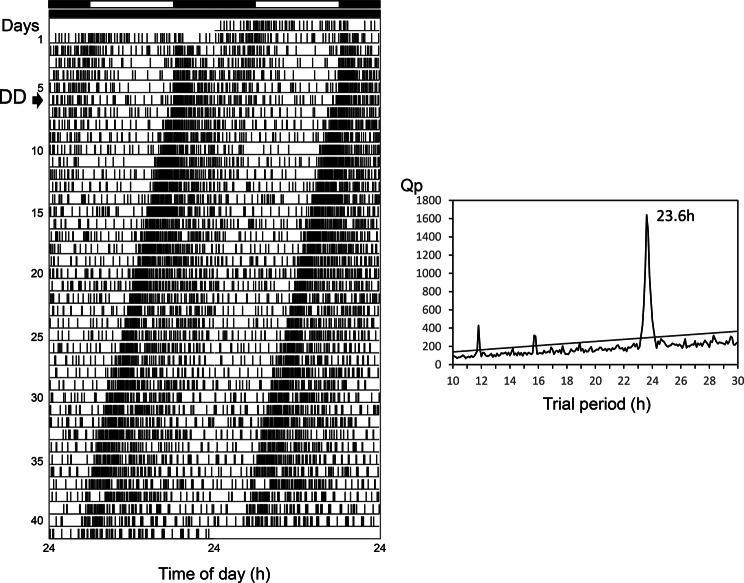
Representative double-plotted actogram (left panel) and corresponding periodogram (right panel) of a control *Gryllus bimaculatus* cricket under a 12:12 light–dark (LD) cycle followed by constant darkness (DD). The transition to DD occurred at 18:00 on day 6, as indicated by the arrow. White and black bars denote the light and dark phases, respectively. The periodogram obtained from days 7 to 41 reveals a free-running circadian rhythm with a period of 23.6 h under DD. The oblique line in the periodogram represents the 0.05% significance threshold

We measured locomotor activity in 12 adult male white-eye control crickets. Figure 2 shows a representative actogram and a chi-square periodogram. Under LD conditions, they showed a nocturnal activity rhythm, with activity concentrated in the first several hours of the dark phase. The nocturnal activity persisted in the ensuing constant darkness (DD) with a period shorter than 24 h. The average free-running period was 23.78 ± 0.12 (mean ± SD) h and the power was 801.93 ± 573.21. These characteristics are quite similar to those in black-eyed wild-type crickets [27].

### Locomotor rhythm of *tim*^KO^ crickets

**Fig. 3:**
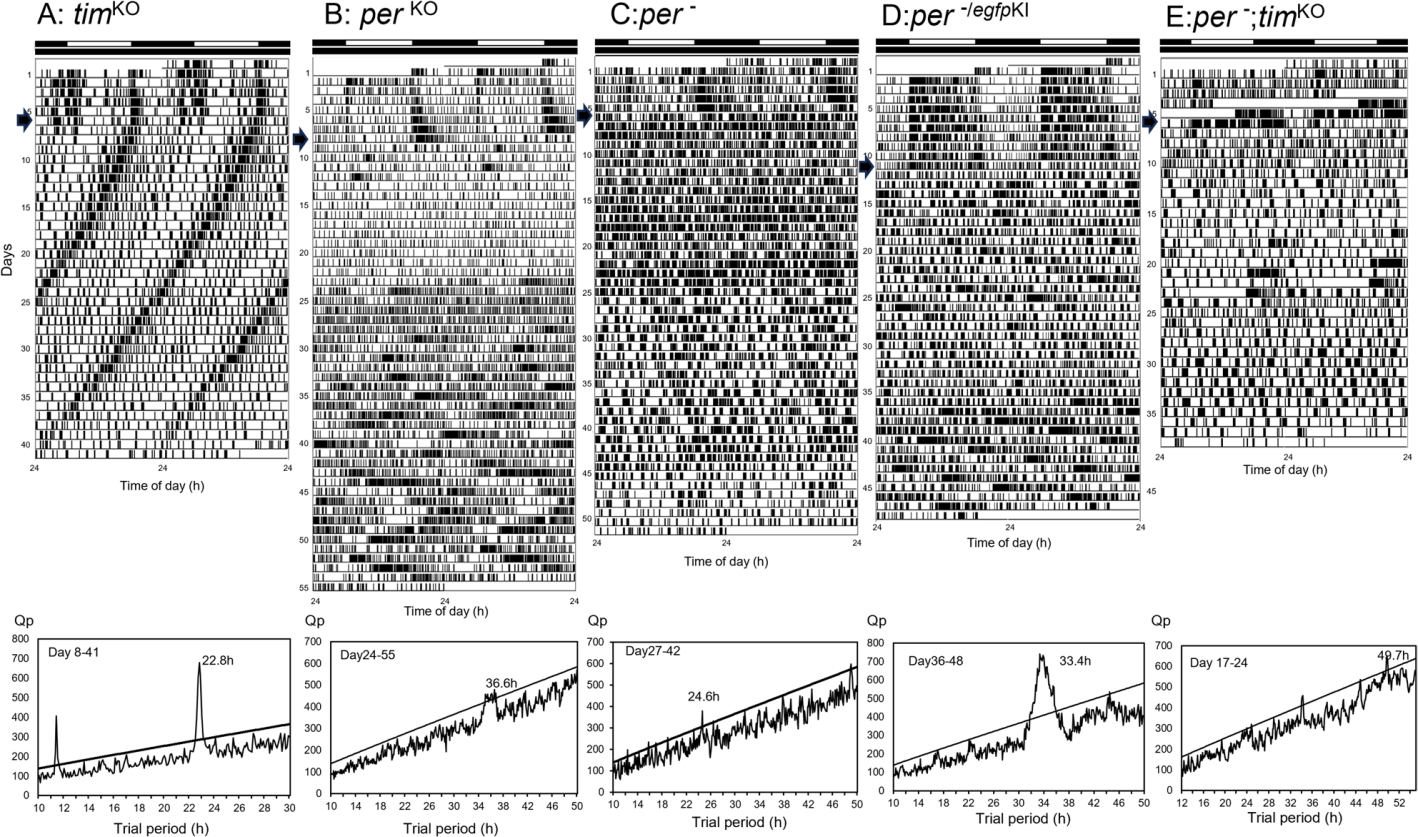
Representative double-plotted actograms (top panels) and corresponding periodograms (bottom panels) of *tim*^KO^ (**A**), *per*^KO^ (**B**), *per*^-^ (**C**), *per*^-/*egfp*KI^ (**D**), and *per*^-^;*tim*^KO^ (**E**) *Gryllus bimaculatus* crickets under a 12:12 light–dark (LD) cycle followed by constant darkness (DD). The transition to DD occurred at 18:00 on the day indicated by the arrow. White and black bars denote the light and dark phases, respectively. The periodograms were calculated for the DD days indicated at the upper-left corner of each panel and the assumed periods are shown at the corresponding peaks. The oblique line in the periodogram represents the 0.05% significance threshold

We measured locomotor activity in 15 adult male *tim*^KO^ crickets, in which a frameshift in exon 3 was caused by an insertion of 5 bases. Figure 3A shows a representative actogram and a chi-square periodogram. Under LD conditions, most crickets showed a bimodal locomotor rhythm with peaks at lights-on and lights-off. On transfer to DD, all the crickets showed a free-running rhythm in which only the lights-off peak persisted with a period shorter than 24 h. The average free-running period was 23.06 ± 0.20 (mean ± SD) h, significantly shorter than that of control crickets (*t*-test, *p* < 0.001). The average power was 707.46 ± 531.67, which was not significantly different from that of control crickets (*t*-test, *p* = 0.6614). The results were quite similar to those obtained in crickets treated with ds*tim* [7].

### Locomotor rhythm of *per*^KO^, *per*^-^, and *per*^-/*egfp*KI^ crickets

We measured locomotor activity in 14 adult male *per*^KO^ crickets, in which a frameshift was caused by an insertion of 17 bases in exon 2. A representative actogram and a chi-square periodogram are shown in Fig. 3B. Under LD conditions, the crickets showed a nocturnal activity rhythm with a peak occurring a few hours after lights-off. When transferred to constant darkness (DD), three crickets showed persistent arrhythmic activity throughout the entire recording period. Among the remaining 11 individuals, two initially exhibited an extremely long free-running period (nearly 48 h), but their rhythms soon disappeared, while the others lost rhythmicity shortly after the shift to DD. Nevertheless, all 11 individuals eventually began to show complex rhythmic patterns after 10–15 days in DD. In the cricket shown in Fig. 3B, the chi-square periodogram revealed a free-running period of 36.6 h. Some crickets showed multiple components running with different free-running periods as exemplified in Supplementary Figure S3. The free-running periods widely varied depending on the individuals, averaging 33.35 ± 10.72 h (*n* = 11), and the power was 73.85 ± 66.00 (*n* = 11). Both were significantly different from those of control crickets (Dunnett’s test, *p* < 0.01).

With 14 *per*^-^ mutant crickets which lacked a large part of the coding region (exon2-10) of the *per* gene we obtained basically similar results to those in *per*^KO^ crickets. Under LD, the crickets exhibited a nocturnal activity rhythm as shown in Fig. 3C. The rhythm diminished in the ensuing DD in all individuals, but after 10–20 days in DD, eight crickets began to show a significant rhythmicity, as detected by the chi-square periodogram. The free-running period considerably varied among individuals, averaging 32.65 ± 9.65 h, and the power was 39.03 ± 16.66. Both were significantly different from those of control crickets (Dunnett’s test, *p* < 0.05 and *p* < 0.01 for the period and power, respectively).

We also measured locomotor activity in 29 *per*^-^ mutant crickets in which *egfp* was knocked into exon 1 of the *per* gene using the CRISPR/Cas9 system. The *per*^-/*egfp*KI^ crickets also showed locomotor rhythms similar to those of *per*^KO^ and *per*^-^ mutant crickets, as shown in Fig. 3D. Under LD, they showed either a diurnal (*n* = 7, exemplified in Fig. 3D) or nocturnal rhythm (*n* = 22). On transfer to DD, 6 crickets showed a rhythm persisting with a period longer than 24 h, which gradually disappeared in prolonged DD. After about 20 days of DD, 22 crickets began to exhibit a rhythm persisting with a period longer than 24 h. The period again varied widely among individuals, averaging 35.44 ± 6.49 h, and the power was 92.61 ± 77.13 (Table 1). Both were significantly different from those of control crickets (Dunnett’s test, *p* < 0.01).

**Table 1:** Summary of the locomotor rhythm analysis under constant darkness (DD) in control (white), *tim*^KO^, *per*^KO^, *per*^-^, *per*^-/*egfp*KI^, and *per*^-^;*tim*^KO^

Strain	Number of animals			Free-running period mean ± SD (h)	Power
	Total, n	Rhythmic, n (%)	Arrhythmic, n (%)		
white	12	12 (100.0%)	0 (0.0%)	23.78 ± 0.12	801.93 ± 573.21
*tim*^KO^	15	15 (100.0%)	0 (0.0%)	23.06 ± 0.20^a^	707.46 ± 531.67
*per*^KO^	14	11 (78.6%)	3 (21.4%)	33.35 ± 10.72^b^	73.84 ± 66.00 ^b^
*per*^-^	14	8 (57.1%)	6 (42.9%)*	32.65 ± 9.65 ^b^	39.03 ± 16.66 ^b^
*per*^-/*egfp*KI^	29	22 (75.9%)	7 (24.1%)	35.44 ± 6.49 ^b^	92.61 ± 77.13 ^b^
*per*^-^;*tim*^KO^	13	8 (61.5%)	5 (38.5%)*	37.00 ± 9.17 ^b^	43.91 ± 18.30 ^b^

*The ratio was significantly greater than in control crickets (*G*-test, *p* < 0.05). ^a^The value is significantly shorter than in the control cricket (*t*-test, *p* < 0.001). ^b^The values were significantly different from those of control crickets (Dunett’s test, *p* < 0.05)

### Effects of *per*^-^ and *tim*^KO^ on the locomotor rhythms

We then measured the locomotor activity of 13 crickets carrying *per*^-^ and *tim*^KO^ mutations (*per*^-^;*tim*^KO^). They showed either nocturnal (*n* = 4), diurnal (*n* = 4), bimodal (*n* = 4), or arrhythmic activity (*n* = 1) under LD conditions. Figure 3E exemplifies an actogram and a chi-square periodogram of a diurnally active cricket. On transfer to DD, five crickets became arrhythmic and no clear rhythm was detected throughout the recording period, while 8 crickets showed a faint but significant rhythm with variable periods, averaging 37.00 ± 9.17 h. Representative actograms and periodograms are shown in Figure 3E and Supplementary Figure S4. The power was 43.91 ± 18.30. Both were significantly different from those of control crickets (Dunnett’s test, *p* < 0.01). Thus, although *per* and *tim* genes play an important role in generating circadian locomotor rhythms, some oscillatory mechanism that functions independently of the *per*/*tim* loop may regulate the residual rhythm.

### Comparison of free-running period and ratio of rhythmic crickets among *per*^KO^, *per*^-^, *per*^-/*egfp*KI^, and *per*^-^;*tim*^KO^ crickets

The free-running periods and ratios of rhythmic and arrhythmic crickets in each strain are shown in Table 1. The ratios of arrhythmic crickets in *per*^-^ and *per*^-^;*tim*^KO^ crickets were significantly higher than those in control crickets (pairwise *G*-test, *p* < 0.01 and *p* < 0.05, respectively), while there was no significant difference among *per*^KO^, *per*^-^, *per*^-/*egfp*KI^, and *per*^-^;*tim*^KO^ crickets (*G*-test, *p* > 0.05).

The free-running period and the power were not significantly different among the *per*^KO^, *per*^-^, *per*^-/*egfp*KI^, and *per*^-^;*tim*^KO^ crickets (ANOVA *F*_3,45_ = 0.49, *p* = 0.689 for the period; *F*_3,45_ = 2.12, *p* = 0.111 for the power).

### Effects of *per*^KO^, *per*^-^, or *tim*^KO^ on expression of clock genes

**Fig. 4:**
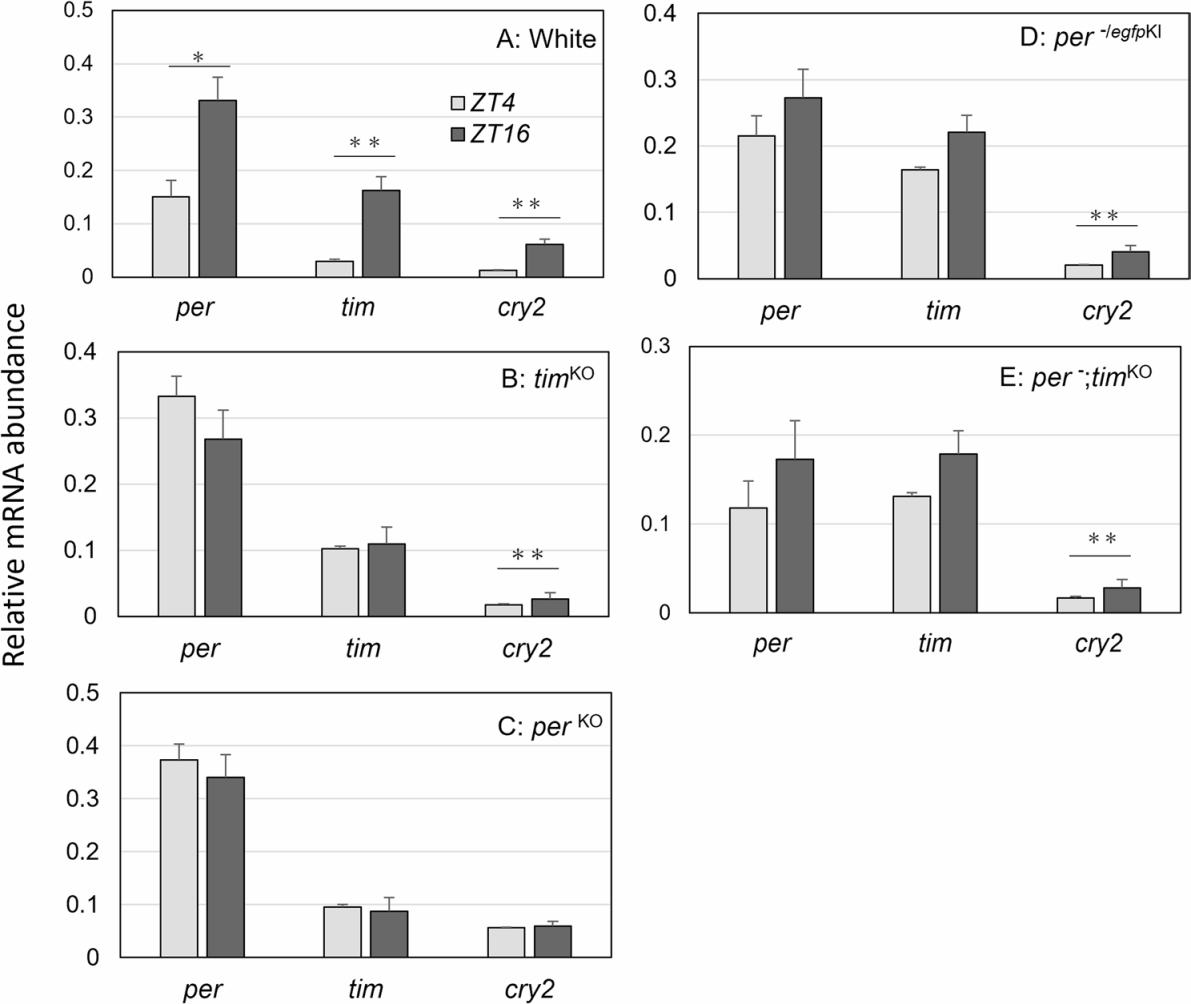
Daily expression of clock genes *per, tim*, and *cry2* in wild-type (**A**), *tim*^KO^ (**B**), *per*^KO^ (**C**), *per*^-/*egfp*KI^ (**D**), and *per*^-^;*tim*^KO^ (**E**) *Gryllus bimaculatus* crickets under a 12:12 light–dark (LD) cycle. The measurement was performed at ZT4 and ZT16. ** *p* < 0.01, * *p* < 0.05, *t*-test

We measured expression levels of *per*, *tim*, and *cry2* in wild-type, *tim*^KO^, *per*^KO^, *per*^-/*egfp*KI^ and *per*^-^;*tim*^KO^ crickets at ZT4 and ZT16 under LD conditions. The results are shown in Fig. 4. Wild-type crickets showed a clear, significant day/night difference in *per*, *tim*, and *cry2* mRNA levels (*t*-test, *p* < 0.05 for *per* and *p* < 0.01 for *tim* and *cry2*), while in crickets carrying *tim*^KO^, *per*^KO^, *per*^-/*egfp*KI^ or *per*^-^;*tim*^KO^, *per* and *tim* lost the day/night changes (*t*-test, *p* > 0.05). However, *cry2* retained a significant day/night difference in its expression in *tim*^KO^, *per*^-/*egfp*KI^, and *per*^-^;*tim*^KO^ crickets (*t*-test, *p* < 0.01), although the magnitude of this difference was reduced.

### Identification of clock cells expressing *per*

**Fig. 5:**
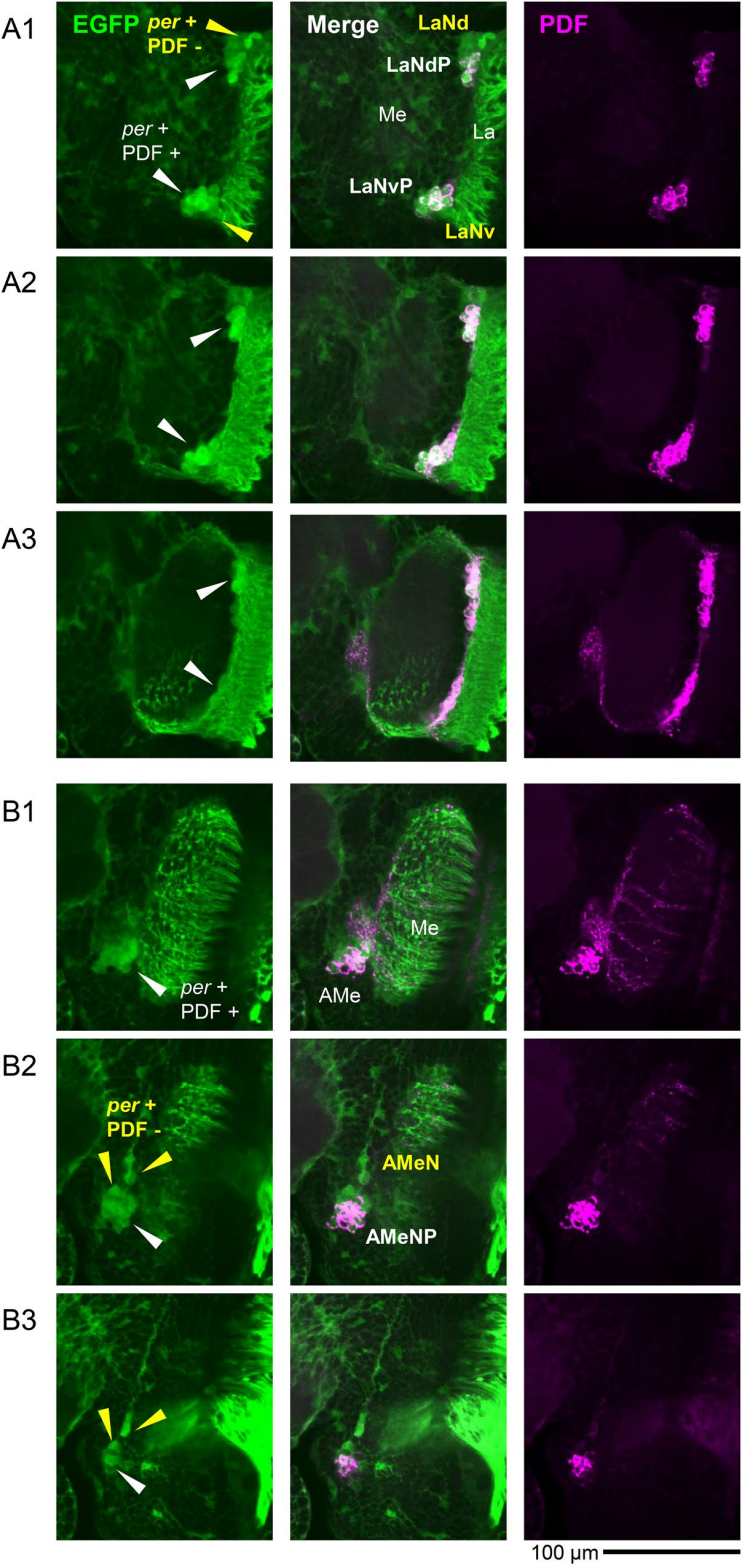
EGFP and PDF immunohistochemistry in the optic lobe (**A, B**) of the first instar nymphal cricket of *per*^-/*egfp*KI^ mutant *Gryllus bimaculatus*. The panels show a frontal view, with the dorsal side oriented toward the top of the image. To account for the thickness of the brain tissue, Z-stack images were processed into maximum intensity projections of three consecutive optical sections. Three representative projections at different depths are presented for both the lamina (A1–A3) and accessory medulla (B1–B3) regions. **A**: Left panel shows EGFP (green) positive (*per*-expressing) cells located on the border between medulla (Me) and lamina (La). Right panel shows PDF (magenta) immunoreactive cells. Middle panel shows merge of EGFP and PDF signals, indicating that most of the *per*-expressing cells also express PDF. These EGFP-positive cells were categorized into four groups: PDF-positive dorsal La neurons (LaNdP), PDF-negative dorsal La neurons (LaNd), PDF-positive ventral La neurons (LaNvP), and PDF-negative ventral La neurons (LaNv). White and yellow arrowheads indicate PDF-positive and PDF-negative *per*-expressing cells, respectively. **B**: Left panel shows EGFP (green) positive (*per*-expressing) cells located close to the accessory medulla (AMe). Right panel shows PDF (magenta) immunoreactive cells. Middle panel shows merge of EGFP and PDF signals, indicating that the more than half of the *per*-expressing cells also express PDF. The EGFP-expressing clusters in this region were designated as PDF-positive AMe neurons (AMeNP) and PDF-negative AMe neurons (AMeN)

We examined the expression of EGFP in first-instar nymphal *per*^-^ crickets carrying *egfp* knocked into exon 1 of *per*. We found three clusters of EGFP-positive cells in the optic lobe (Fig. 5A, B). One was located in the proximal ventral region close to the accessory medulla (Fig. 5B). The cluster consisted of ~ 20 cells; some of them were apparently PDF-positive and some were PDF-negative. We designated these as PDF-positive accessory medulla (AMe) neurons (AMeNP) and PDF-negative AMe neurons (AMeN). Their neural processes were also labeled by EGFP, distributing over the frontal surface of the medulla. The other two clusters were located on the dorsal and ventral border, respectively, between the medulla and lamina neuropils (Fig. 5A). We named these clusters as follows: PDF-positive dorsal La neurons (LaNdP), PDF-negative dorsal La neurons (LaNd), PDF-positive ventral La neurons (LaNvP), and PDF-negative ventral La neurons (LaNv). In these EGFP‑positive cells, EGFP signals were detected in both the nucleus and the cytoplasm. The cell counts for each cluster are summarized in Supplementary Table S4 (*n* = 7 optic lobes). Cell size was approximately 6 μm across all populations, with no differences observed between cell groups. In the cerebral lobe, a cluster of 3 cells showed strong EGFP expression in the mid-lateral region of each hemisphere (Supplementary Figure S5). In addition, there are many cells weakly expressing EGFP, distributing over the cerebral lobe (Supplementary Figure S5).

## Discussion

### Role of *per* and *tim* in the cricket’s circadian clock

The clock gene *per* has been generally accepted to be the most important and essential component of the oscillatory mechanism. The present study showed that a substantial proportion of the crickets without functional *per* gene still exhibited circadian rhythms in their locomotor activity but with varying free-running periods (Fig. 3B–E and Supplementary Figure S3, Table 1). Although the possibility of additional background mutations in the *per*^*−*^ strain cannot be ruled out, its phenotype closely resembles that of the *per*^KO^ strain generated by targeted genome editing. This observation suggests that the phenotype of the *per*^*−*^ strain is most likely attributable to disruption of *per* rather than to unrelated background mutations. The results provide compelling evidence for an oscillatory mechanism capable of generating rhythms in the absence of *per*, though the period is variable. Supporting this view, studies in other hemimetabolous insects have shown that RNAi of *per* did not eliminate locomotor rhythms in most treated cockroaches *Rhyparobia maderae* [28]. The existence of *per*-less oscillation has been suggested in holometabolous insects. In the fruit fly *Drosophila melanogaster*, *per*^0^ mutants exhibit locomotor rhythms only under photoperiods or thermoperiods within the circadian range [4, 5, 29], suggesting that these rhythms are driven by an endogenous circadian oscillator. Additional evidence comes from daily endocuticle deposition rhythms in *Drosophila*, which were observed exclusively under photoperiods within a circadian range [3]. Taken together, these findings suggest that circadian oscillations independent of the *per* gene may be widespread among insects.

However, the rhythms in *per*^KO^ and *per*^-^ crickets were generally weak, often manifesting as arrhythmic or showing low‑amplitude, complex patterns, for which two explanations are possible. One is that the *per*-less oscillatory loop produces only weak oscillation. The other is that the rhythms in individual clock neurons desynchronize and run at different free-running periods, like mammalian SCN clock neurons kept under constant light [30]. The latter hypothesis may also explain why some crickets exhibit weak or complex rhythms only after a certain period of arrhythmicity under constant darkness. During the prolonged constant darkness, however, some desynchronized cells may become synchronized through some intercellular communication to produce a rhythm, as known for mammalian SCN circadian oscillatory neurons [31]. In addition, we found that some *per*^-^ crickets exhibited diurnal activity under LD (Fig. 3D). Although the mechanism underlying this daytime activity remains unclear, it may be attributable to a direct or masking effect of light, as the rhythmicity disappeared immediately after transfer to DD (Fig. 3D). Light-driven daily activity has also been reported in the arrhythmic *per*^0^ mutant of *Drosophila* [32]. Thus, it seems likely that light can influence behavior when the driving force of the circadian clock is weakened.

*tim* is an essential component in the circadian clock machinery in *Drosophila*, where lack of functional *tim* results in loss of locomotor rhythms [33]. In contrast, we found that *tim*^KO^ crickets showed a clear rhythm but the free-running period under DD was significantly shorter than that of the control crickets. A similar result was obtained in the linden bug *Pyrrhocoris apterus* [34]. A comparable shortening of the free-running period was also reported for crickets and cockroaches treated with *tim*^RNAi^ [7, 28]. These findings suggest that unlike in *Drosophila*, *tim* is not an essential component in the clock machinery but plays a role in determining the free-running period in hemimetabolous insects including crickets, cockroaches, and linden bugs.

In this study, nearly 60% of the double mutants of *per*^-^;*tim*^KO^ resulted in a loss of circadian locomotor rhythm. This suggests that *per* and *tim* work together in the cricket clock and most likely form a negative feedback loop that plays an important role in producing a circadian oscillation, as suggested previously [1, 35]. However, a weak but significant rhythm was detected in more than 40% of the *per*^-^;*tim*^KO^ double mutant crickets. This strongly suggests the existence of an oscillatory mechanism that functions independently of the *per*/*tim*-loop. The circadian rhythm in the double mutant crickets is most likely generated by a *cry*-based loop. This assumption is based on our previous results that *cry2* maintains its rhythmic expression even after *tim*^RNAi^ [6]. Although rhythmic expression needs to be accurately examined at sufficient time points under both LD and DD conditions, the present results can be interpreted as providing tentative support for daily rhythmic expression of *cry2* in *per*^-/*egfp*KI^, *tim*^KO^, and *per*^-^;*tim*^KO^ double mutant crickets. In *per*^KO^ crickets, significant daily changes were not found in *cry2* expression. Although the cause of this arrhythmic expression of *cry2* remains unclear, it may reflect an asynchrony among the clock neurons associated with the initial arrhythmia of locomotor activity exhibited in the majority of the *per*^KO^ crickets upon transfer to DD.

In the cricket, the *cry*-based oscillatory loop consists of CRY2 variants and CRY1 [6]. The present study shows that oscillatory activity in the *cry*-based loop is rather weak and unstable: a loss of the *per*/*tim*-loop caused by a double null mutation of *per* and *tim* leads to either a loss of locomotor rhythm or a weakened rhythm with extremely long free-running periods (Fig. 3E, Table 1). In crickets with *cry1* and *cry2* double knockdown, circadian locomotor rhythm persists with variable free-running periods and mRNA of *per* and *tim* shows rhythmic expression [6], suggesting that the oscillation of the *per*/*tim*-loop alone is rather labile. These facts suggest that a stable circadian rhythm requires the coordinated function of both the *per*/*tim*-loop and the *cry*-based loop.

### Anatomical localization of the circadian clock

In the cricket *G. bimaculatus*, the clock location was investigated by neuroethological analysis and the optic lobe has been identified as the clock tissue [8, 11]. However, no clear cellular localization of the clock is available. Here, we employed the CRISPR/Cas9 system to knock the *egfp* gene into the first exon of *per* to visualize its expression. EGFP-expressing cells were located within or adjacent to the PDF-positive cell clusters at the proximal base of the medulla and along the dorsal and ventral borders between the lamina and medulla neuropils (Fig. 5). The distribution of EGFP was consistent with previous in situ hybridization results for *per* and *cry* mRNA, at least in the outer lamina region [36]. These findings suggest that our *egfp* knock-in effectively captures the regulatory landscape of the *per* locus, with EGFP expression accurately reflecting the endogenous spatial distribution of *per*. The importance of PDF in the generation of circadian locomotor rhythms has been suggested in crickets and cockroaches [37–39], and our present results are consistent with those reports. Intriguingly, holometabolous insects, such as *Drosophila melanogaster* [40] and *Apis mellifera* [41, 42], lack clock neurons in the lamina, whereas hemimetabolous insects, including *G. bimaculatus* and *Acyrthosiphon pisum* [43], possess them. The loss of lamina clock neurons during evolution may be attributed to changes in brain structure and reorganization of light-input pathways in holometabolous insects.

In *Drosophila* clock neurons, PER is known to be rhythmically expressed: it is localized in the cytoplasm in the early night, translocates to the nucleus late at night, and disappears in the early day [44, 45]. In our study, PER was detected in both the cytoplasm and the nucleus, likely because the *per*^‑^ genetic background results in arrhythmic or weakly rhythmic phenotypes (Fig. 3D). Whether PER cycles in a manner similar to *Drosophila* remains to be determined in future studies.

Axonal processes of some medulla PDF neurons in *G. bimaculatus* extend toward the border of the lamina and medulla and then turn to project to the central brain [24]. Since removal of the outer part of the medulla abolishes circadian locomotor rhythms [46], these neurons and/or lamina clock neurons are likely to play a crucial role in rhythm generation. The medulla clock neurons may regulate locomotor rhythms either directly through their projections to the brain or indirectly by modulating neural activity in the optic lobe, which is transmitted to the cerebral lobe and influences locomotor behavior. The lamina clock neurons are likely to act through the latter pathway, as their projections are confined to the optic lobe [24]. These explanations are based on the fact that neuronal activity originating in the optic lobe and propagating to the cerebral lobe exhibits a clear circadian rhythm [10, 11]. The critical question to be elucidated is how such neural information governs circadian locomotor rhythms.

Additionally, *egfp*-KI revealed that in the cerebral lobe, there is a cluster of cells strongly expressing EGFP and widely distributed cells weakly expressing EGFP. The role of these cells is currently unknown, but these cells may contribute to generating circadian locomotor rhythms. There are several lines of evidence suggesting the contribution of the extra-optic lobe clock in the regulation of behavioral rhythms. It is known that many crickets show residual circadian locomotor rhythms for several days after optic lobe removal [47], that some crickets show a weak circadian rhythm when the neural connection is regenerated between the retinae and the optic stalk after the optic lobe removal [48], and that singing rhythms of optic-lobeless crickets were entrained only to temperature cycles with a period close to 24 h [49]. The functional contribution of these cerebral *per*-expressing cells to circadian rhythm regulation has yet to be determined.

## Conclusions

This study reveals that in the cricket *G. bimaculatus*, *tim* plays a central role in determining the free-running period, whereas loss of *per* disrupts canonical rhythmicity and generates complex oscillations with unusually long free-running periods. Remarkably, nearly 40% of *per*^−^;*tim*^KO^ double mutants retained weak but significant circadian rhythms and continued to exhibit what appears to be daily rhythmic expression of *cry2*, despite the loss of rhythmic *per* and *tim* expression. These findings suggest the possible existence of a *cry*‑based oscillatory mechanism operating independently of the classical *pe*r/*tim* feedback loop. Moreover, the identification of three *per*‑reporter–positive cell clusters in the optic lobe points to a structured clock network underlying locomotor rhythms. Together, these results challenge the current model of the insect circadian system and lay the groundwork for uncovering both the *per*/*tim*‑less oscillator and the functional organization of the circadian clock network.

## Electronic supplementary material

Below is the link to the electronic supplementary material.

Supplementary Material 1. Supplementary Figure S1. Sequencing analyses of the *per*^-^ transcript. Sequence analysis of the RT-PCR product from the *per*^-^ strain (Fig. 1D) showed that the amplicon generated using the *per*-Fw and *per*-Rv primers is markedly shorter than the size predicted for wild-type *per* (2,490 bp; AB375516), indicating a deletion spanning exons 2 through 10, which encode the PAS domain. The two PCR products of different sizes differed in the length of the untranslated region transcribed from exon 1

Supplementary Material 2. Supplementary Figure S2. Sequencing analyses of the *per*^-/*egfp*KI^ strain. Sequence analysis of both ends of the PCR product (Fig. 1F) showed that the expression cassette was inserted in the forward orientation in the *per*^-/*egfp*KI^ strain. Black text indicates partial sequences of the *per* region, while green text indicates partial sequences of the inserted cassette. A few base insertions and deletions were confirmed at the junction

Supplementary Material 3. Supplementary Figure S3. An example of locomotor activity record (left panel) and chi-square periodogram of adult male *per*^KO^*Gryllus bimaculatus* showing multiple free-running components. The transition to DD occurred at 18:00 on day 8, as indicated by an arrow. White and black bars denote the light and dark phases, respectively. The periodogram obtained from days 21 to 46 reveals rhythmic components free-running with periods of 26.9 h and 29.7 h under DD. The oblique line in the periodogram represents the 0.05% significance threshold

Supplementary Material 4. Supplementary Figure S4. Representative double-plotted actograms (top panels) and corresponding periodograms (bottom panels) of *per*^-^;*tim*^KO^*Gryllus bimaculatus* crickets under a 12:12 light–dark (LD) cycle followed by constant darkness (DD). Panels **A-C** show rhythmic individuals, whereas panel D shows an arrhythmic individual. The transition to DD occurred at 18:00 on the day indicated by the arrow. White and black bars denote the light and dark phases, respectively. The periodograms were calculated for the DD days indicated in the upper-left corner of each panel and the assumed periods are shown at the corresponding peaks. The oblique line in each periodogram represents the 0.05% significance threshold

Supplementary Material 5. Supplementary Figure S5. EGFP immunohistochemistry in the left half of the brain of the first instar nymphal cricket of *per*^-/*egfp*KI^ mutant *Gryllus bimaculatus*. The panel shows a frontal view, with the dorsal side oriented toward the top of the image. EGFP is strongly expressed in 3 cells in the mid lateral region of the protocerebrum (arrowhead) and weakly expressed in many cells. Small arrows indicate the non-specifically stained tracheae. CL, cerebral lobe; Me, medulla; La, lamina

Supplementary Material 6. Supplementary Table S1. Target sequence of guide RNAs used in this study

Supplementary Material 7. Supplementary Table S2. Primers used for sequencing

Supplementary Material 8. Supplementary Table S3. PCR primers used for quantitative RT-PCR

Supplementary Material 9. Supplementary Table 4. Numbers of *per-egfp*-expressing cells in the optic lobe

## Data Availability

The datasets used and/or analysed during the current study are available from the corresponding author on reasonable request.

## Abbreviations

AMe: Accessory medulla
AMeN: PDF-negative accessory medulla neurons
AMeNP: PDF-positive AMe neurons
CLK: CLOCK
*cry*: *cryptochrome*
*cry2*: *cryptochrome2*
CYC: CYCLE
DD: Constant darkness
EGFP: Enhanced green fluorescent protein
La: Lamina
LaNd: PDF-negative dorsal La neurons
LaNdP: PDF-positive dorsal La neurons
LaNv: PDF-negative ventral La neurons
LaNvP: PDF-positive ventral La neurons
LD: Light-dark cycle
*per*: *period*
*tim*: *timeless*
TTFL: Transcriptional translational feedback loop
