# Supplementary material for: Knockout analysis of period and timeless and EGFP-based visualization of per-expressing clock cells in the cricket circadian clock
Source: Zoological Lett. 2026 Jul 7;12:12. doi: 10.1186/s40851-026-00267-6 (PMC13360532; doi:10.1186/s40851-026-00267-6)
Supplement: Supplementary file 1 — Supplementary Material 1. Supplementary Figure S1. Sequencing analyses of the per- transcript. Sequence analysis of the RT-PCR product from the per- strain (Fig. 1D) showed that the amplicon generated using the per-Fw and per-Rv primers is markedly shorter than the size predicted for wild-type per (2,490 bp; AB375516), indicating a deletion spanning exons 2 through 10, which encode the PAS domain. The two PCR products of different sizes differed in the length of the untranslated region transcribed from exon 1 [file 40851_2026_267_MOESM1_ESM.pdf]

- The larger PCR product in Fig.1D (1,359bp)

AGTCGCGTCTACAACAAGCTTTGGTTCGTGTGTGCGCGTTCTCTGGCACCTGGACGACGCGT
GTGAGACGCAAGTGTGAACCGGGAAGACGAGCAGAAATGTGAGTACGCCTTCTCCGAGTGT
GCTGTGCCGCGGTGTATTGTGACACGGTGTAGTGTGACAGTGGCCCAAAATTAATTTTCTAG
TCTCTTTTTTTTTGTCCTTTCTCCTAAGTGTGACAGTGCATTGCGAGTTCTTTCAGTGCTGGTTA
GTACTTGAAGAAGCTACGCCGTTGTGAACGAATATTCACAGCTGCGTATTATCTGTTGGTTTTT
TTTGTGAAGTACAGTGAAAATGAACAGATAAACTATGTTCTTTGAATCAAACGTCTTGTGTTG
AACCGTCTAACAAAGGTGTCACAAACCTTTGATCGTATATAGCTGCTGTCCTTGTTAGTCCGTG
AGCCATGCCTGTTCGTGACGACACGACAAAGACAATACAACGAACAACAGAAGCAGCTAAG
CAAGTGGCATCTAAACGCTGTAAAGATTTGGCTACATTCATGGAGATTTTAATGGATGAGGTCA
CAAAGCCTGAACTGAAAGTAGATCTGCCATCAGAAGAACAAAGCTTCTCTAAGAACATTATTT
TGCAGGAACGAGACTCTGTCATGTTGGGTGAAATTTCACCCCATCATGACTATTATGATAGTAA
ATCTTCATCTGAAACTCCTCCGAGCTATAATCAACTTAACTATAATGAAAACATCCAAAGATTC
TTTGAAAGTAAACCCAAAACAACGCTTTCTGATGAATCTGGTGAATCGAAGACAGATGCCAA
TCGATCCCACAACAGCACTGATGAGGAGGGGAAGAGTATGCCTGTTGCAGATTCCAGTCTCA
ATTCAAGCAACAGGAAATGTTGTTCTCCTGTCAACGGAAGTGGGAGTGGCAGTGGTGGGAGC
TCTGGCAGTGCTGGGATGCCGGGCAGCGCTGCAAGCCGTGGAGATACCAGCGCCACCAACAC
CTCTCATGGATCCTACAAACCACCACATCTTACTGAGGCTCTTCTTTGCAGACATAATGAAGAC
ATGGAGAAGCAAATGGTACAGAAACATCGAGAACTAAGATCAAAAGGGGACAGTAAGAAAA
AGATGTCTCATGAGAAACTTCAGGAACAGAACCATGGTGTAAAGCGAAGTGGATCTCACTCA
TGGGAGGGCGAACCCTTCAAAGCAAGCAAGCATCCTCATGTCGAAAACCTGCTGGCTTCAGG
GAATGCAGTCCCAATGCCCAATGTGGCTGCGCTCGGAGGTGCAACTCAGATGTCTCCGATGTA
CCCAGGATCTCCAAACGTTAACTTGTGGCCTCCTTTCTCAG

- The smaller PCR product in Fig.1D (968bp)

AGTCGCGTCTACAACAAGCTTTGGTTCGTGTGTGCGCGTTCTCTGGCACCTGGACGACGCGT
GTGAGACGCAAGTGTGAACCGGGAAGACGAGCAGAAATACAATACAACGAACAACAGAAGC
AGCTAAGCAAGTGGCATCTAAACGCTGTAAAGATTTGGCTACATTCATGGAGATTTTAATGGAT
GAGGTCACAAAGCCTGAACTGAAAGTAGATCTGCCATCAGAAGAACAAAGCTTCTCTGAACG
AGACTCTGTCATGTTGGGTGAAATTTCACCCCATCATGACTATTATGATAGTAAATCTTCATCTG
AAACTCCTCCGAGCTATAATCAACTTAACTATAATGAAAACATCCAAAGATTCTTTGAAAGTAA
ACCCAAAACAACGCTTTCTGATGAATCTGGTGAATCGAAGACAGATGCCAATCGATCCCACA
ACAGCACTGATGAGGAGGGGAAGAGTATGCCTGTTGCAGATTCCAGTCTCAATTCAAGCAAC
AGGAAATGTTGTTCTCCTGTCAACGGAAGTGGGAGTGGCAGTGGTGGGAGCTCTGGCAGTGC
TGGGATGCCGGGCAGCGCTGCAAGCCGTGGAGATACCAGCGCCACCAACACCTCTCATGGAT
CCTACAAACCACCACATCTTACTGAGGCTCTTCTTTGCAGACATAATGAAGACATGGAGAAGC
AAATGGTACAGAAACATCGAGAACTAAGATCAAAAGGGGACAGTAAGAAAAAGATGTCTCAT
GAGAAACTTCAGGAACAGAACCATGGTGTAAAGCGAAGTGGATCTCACTCATGGGAGGGCG
AACCCTTCAAAGCAAGCAAGCATCCTCATGTCGAAAACCTGCTGGCTTCAGGGAATGCAGTC
CCAATGCCCAATGTGGCTGCGCTCGGAGGTGCAACTCAGATGTCTCCGATGTACCCAGGATCT
CCAAACGTTAACTTGTGGCCTCCTTTCTCAG

Supplementary Figure S1. Sequencing analyses of the *per*⁻ transcript.

Sequence analysis of the RT-PCR product from the *per*⁻ strain (Fig. 1D) showed that the amplicon generated using the *per*-Fw and *per*-Rv primers is markedly shorter than the size predicted for wild-type *per* (2,490 bp; BAG48878), indicating a deletion spanning exons 2 through 10, which encode the PAS domain. The two PCR products of different sizes differed in the length of the untranslated region transcribed from exon 1.
