# Supplementary material for: Knockout analysis of period and timeless and EGFP-based visualization of per-expressing clock cells in the cricket circadian clock
Source: Zoological Lett. 2026 Jul 7;12:12. doi: 10.1186/s40851-026-00267-6 (PMC13360532; doi:10.1186/s40851-026-00267-6)
Supplement: Supplementary file 2 — Supplementary Material 2. Supplementary Figure S2. Sequencing analyses of the per-/egfpKI strain. Sequence analysis of both ends of the PCR product (Fig. 1F) showed that the expression cassette was inserted in the forward orientation in the per-/egfpKI strain. Black text indicates partial sequences of the per region, while green text indicates partial sequences of the inserted cassette. A few base insertions and deletions were confirmed at the junction [file 40851_2026_267_MOESM2_ESM.pdf]

- The junction sequence between *per* and the expression cassette (promoter side)

…TCTCTGGCACCTGGACGACGCGTGTGAGACGCAAGTGTGCTAAATGTCGCCTGGGACATCC
insertion
TGTCCCCCCAGTTCCAGTACGGCTCCAAGAGATCTAGTTCCTT…

- The junction sequence between the expression cassette (*egfp* side) and *per*

…GAGTTCATGCGCTTCAAGGTGCGCATGGAGGGCACCGTGAACGGCCACGAGTTCGAGATC
GAGGGCGAGGGCGAGGGCCGCCCCTACGAGGGCCACAACACCGTGAAGCTGAAGGTGACC
AAGGGCGGCCCCCTGCC(2bp del)(4bp del)GGGAAGACGAGCCGGGAAGACGAGCAGAAATG
TGAGTACGCCTTCTCCGAGTGTGCTGTGCCGCGGTGTATTGTGACACGGTG…

Supplementary Figure S2. Sequencing analyses of the *per*$^{-/egfp\mathrm{KI}}$ strain.

Sequence analysis of both ends of the PCR product (Figure 1F) showed that the expression cassette was inserted in the forward orientation in the *per*$^{-/egfp\mathrm{KI}}$ strain. Black text indicates partial sequences of the *per* region, while green text indicates partial sequences of the inserted cassette. A few base insertions and deletions were confirmed at the junction.
