# Supplementary material for: Knockout analysis of period and timeless and EGFP-based visualization of per-expressing clock cells in the cricket circadian clock
Source: Zoological Lett. 2026 Jul 7;12:12. doi: 10.1186/s40851-026-00267-6 (PMC13360532; doi:10.1186/s40851-026-00267-6)
Supplement: Supplementary file 3 — Supplementary Material 3. Supplementary Figure S3. An example of locomotor activity record (left panel) and chi-square periodogram of adult male perKOGryllus bimaculatus showing multiple free-running components. The transition to DD occurred at 18:00 on day 8, as indicated by an arrow. White and black bars denote the light and dark phases, respectively. The periodogram obtained from days 21 to 46 reveals rhythmic components free-running with periods of 26.9 h and 29.7 h under DD. The oblique line in the periodogram represents the 0.05% significance threshold [file 40851_2026_267_MOESM3_ESM.pdf]

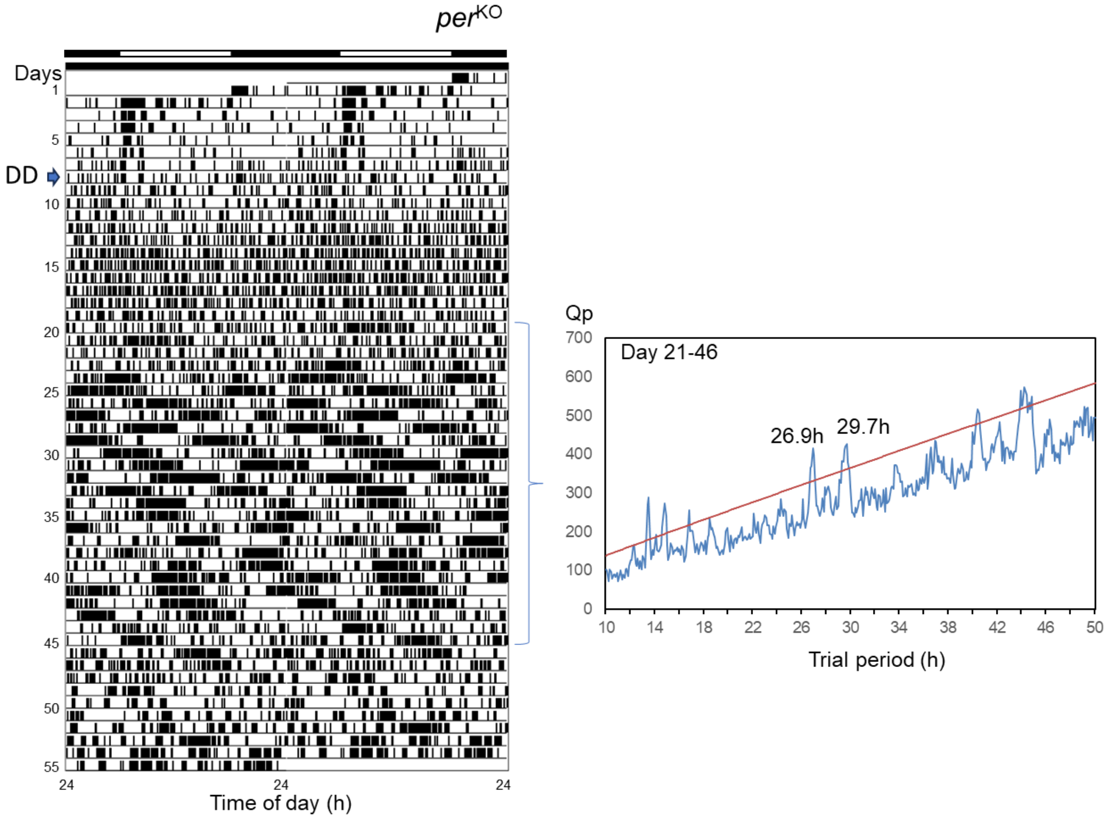

Supplementary Figure S3. An example of locomotor activity record (left panel) and chi-square periodogram of adult male *per*^{KO} *Gryllus bimaculatus* showing multiple free-running components. The transition to DD occurred at 18:00 on day 8, as indicated by an arrow. White and black bars denote the light and dark phases, respectively. The periodogram obtained from days 21 to 46 reveals rhythmic components free-running with a period of 26.9 h and 29.7 h under DD. The oblique line in the periodogram represents the 0.05% significance threshold.
