# Supplementary material for: Knockout analysis of period and timeless and EGFP-based visualization of per-expressing clock cells in the cricket circadian clock
Source: Zoological Lett. 2026 Jul 7;12:12. doi: 10.1186/s40851-026-00267-6 (PMC13360532; doi:10.1186/s40851-026-00267-6)
Supplement: Supplementary file 4 — Supplementary Material 4. Supplementary Figure S4. Representative double-plotted actograms (top panels) and corresponding periodograms (bottom panels) of per-;timKOGryllus bimaculatus crickets under a 12:12 light–dark (LD) cycle followed by constant darkness (DD). Panels A-C show rhythmic individuals, whereas panel D shows an arrhythmic individual. The transition to DD occurred at 18:00 on the day indicated by the arrow. White and black bars denote the light and dark phases, respectively. The periodograms were calculated for the DD days indicated in the upper-left corner of each panel and the assumed periods are shown at the corresponding peaks. The oblique line in each periodogram represents the 0.05% significance threshold [file 40851_2026_267_MOESM4_ESM.pdf]

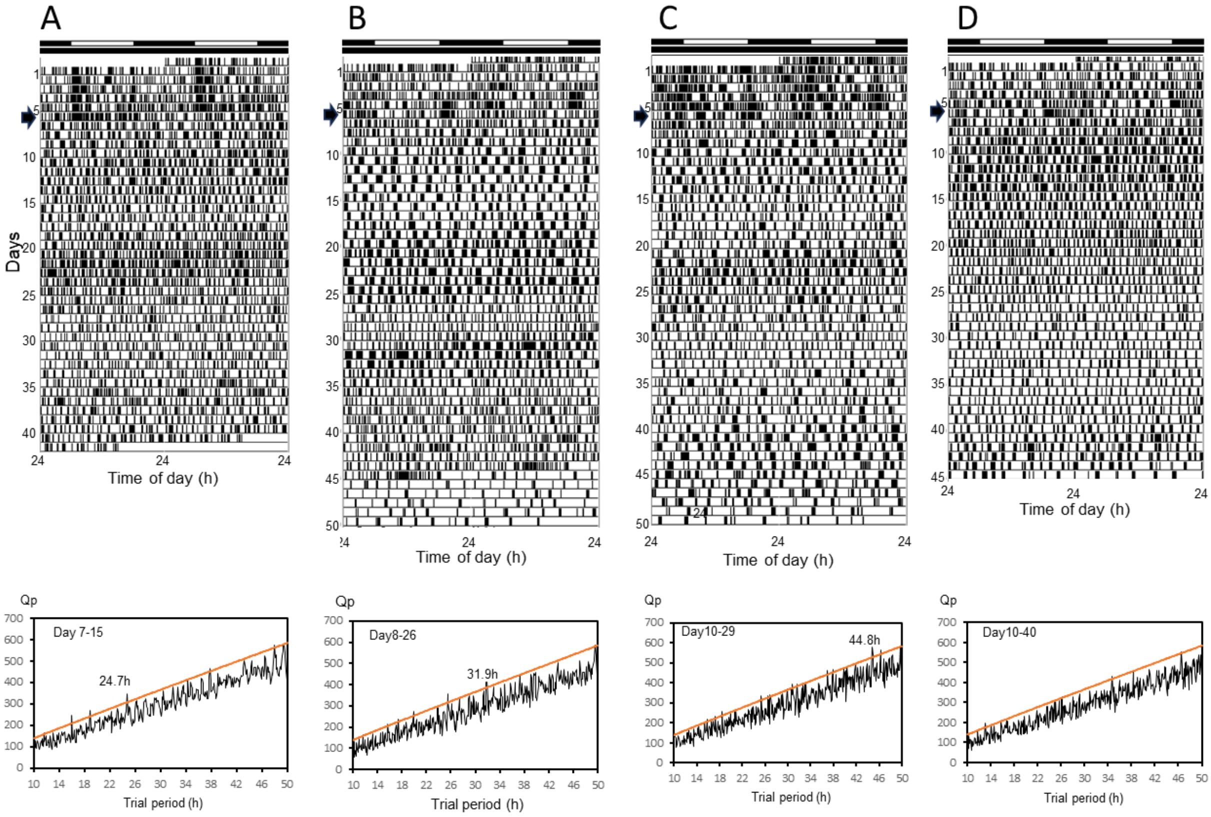

Supplementary Figure S4. Representative double-plotted actograms (top panels) and corresponding periodograms (bottom panels) of *per*$^{-}$;*tim*^{KO} *Gryllus bimaculatus* crickets under a 12:12 light–dark (LD) cycle followed by constant darkness (DD). Panels A-C show rhythmic individuals, whereas panel D shows an arrhythmic individual. The transition to DD occurred at 18:00 on day 6 indicated by the arrow. White and black bars denote the light and dark phases, respectively. The periodograms were calculated for the DD days indicated in the upper-eft corner of each panel and the assumed periods are shown at the corresponding peaks. The oblique line in each periodogram represents the 0.05% significance threshold.
