# Supplementary material for: Knockout analysis of period and timeless and EGFP-based visualization of per-expressing clock cells in the cricket circadian clock
Source: Zoological Lett. 2026 Jul 7;12:12. doi: 10.1186/s40851-026-00267-6 (PMC13360532; doi:10.1186/s40851-026-00267-6)
Supplement: Supplementary file 5 — Supplementary Material 5. Supplementary Figure S5. EGFP immunohistochemistry in the left half of the brain of the first instar nymphal cricket of per-/egfpKI mutant Gryllus bimaculatus. The panel shows a frontal view, with the dorsal side oriented toward the top of the image. EGFP is strongly expressed in 3 cells in the mid lateral region of the protocerebrum (arrowhead) and weakly expressed in many cells. Small arrows indicate the non-specifically stained tracheae. CL, cerebral lobe; Me, medulla; La, lamina [file 40851_2026_267_MOESM5_ESM.pdf]

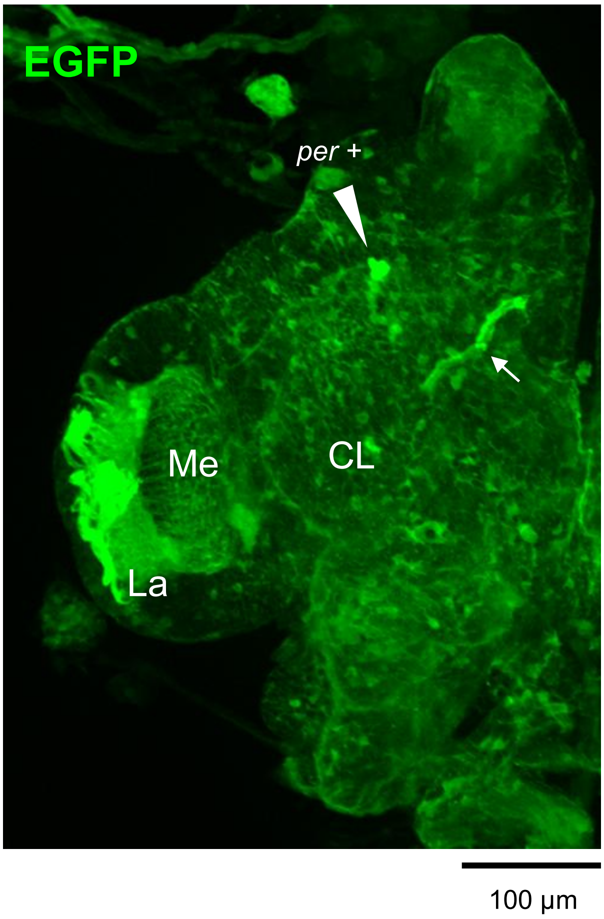

Supplementary Figure S5. EGFP immunohistochemistry in the left half of the brain of the first instar nymphal cricket of *per*$^{-/egfp\mathrm{KI}}$ mutant *Gryllus bimaculatus*. The panel shows a frontal view, with the dorsal side oriented toward the top of the image. EGFP is strongly expressed in 3 cells in mid lateral region of the protocerebrum (arrowhead) and weakly expressed in many cells. Small allow indicate the unspecific stained trachea. CL, cerebral lobe; Me, medulla; La, lamina.
