# Supplementary material for: Knockout analysis of period and timeless and EGFP-based visualization of per-expressing clock cells in the cricket circadian clock
Source: Zoological Lett. 2026 Jul 7;12:12. doi: 10.1186/s40851-026-00267-6 (PMC13360532; doi:10.1186/s40851-026-00267-6)
Supplement: Supplementary file 6 — Supplementary Material 6. Supplementary Table S1. Target sequence of guide RNAs used in this study [file 40851_2026_267_MOESM6_ESM.pdf]

Supplementary Table S1. Target sequence of guide RNAs used in this study.

gRNA name	Target sequence (5' to 3')
per-exon1-gRNA	GTGAGACGCAAGTGTGAACCGGG
per-exon2-gRNA	AGACTGGCTATTGCAGCTGTTGG
tim-exon3-gRNA	CTCTGTGGGAGTTCAGGTTGGGG
DsRed-gRNA	GGATGTCCCAGGCGAAGGGCAGG

The underlined bases indicate the protospacer adjacent motif sequence.
