# Supplementary material for: Knockout analysis of period and timeless and EGFP-based visualization of per-expressing clock cells in the cricket circadian clock
Source: Zoological Lett. 2026 Jul 7;12:12. doi: 10.1186/s40851-026-00267-6 (PMC13360532; doi:10.1186/s40851-026-00267-6)
Supplement: Supplementary file 7 — Supplementary Material 7. Supplementary Table S2. Primers used for sequencing [file 40851_2026_267_MOESM7_ESM.pdf]

Supplementary Table S2. Primers used for sequencing.

Primer name	Primer sequence (5’ to 3’)
Genomic PCR	
per-exon1-Fw	CAGTCGCGTCTACAACAAGC
per-intron1-Rv	TGAAAGAACTCGCAATGCAC
per-exon2-Fw	TGGAGGAAAGTGATACAAGTACCC
per-exon3-Rv	CCACTTGAATTGCTGTGGTG
per-intron1-Fw1	CCTCAAGCCTAAATGACGCC
per-intron1-Rv1	CCGTCAAGAGTGGTTTTGCA
per-intron2-Fw2	ACCGACTTCCTCTCTGCTTC
per-intron2-Rv2	ATTTTGAGCCGGGAATCAGC
per-exon10-Fw	TGTGACACAAACCTCTGAAGA
per-exon11-Rv	AGGCTTTGTGACCTCATCCA
tim-exon3-Fw	CACCATTTGCCCAATGTCTA
tim-exon3-Rv	ACTGCTGGATCATGGAATGG
RT-PCR	
per-RT-Fw*	CAGTCGCGTCTACAACAAGC
per-RT-Rw	CTGAGAAAGGAGGCCACAAG

*It has the same sequence as *per*-exon1-Fw.
