# Supplementary material for: Knockout analysis of period and timeless and EGFP-based visualization of per-expressing clock cells in the cricket circadian clock
Source: Zoological Lett. 2026 Jul 7;12:12. doi: 10.1186/s40851-026-00267-6 (PMC13360532; doi:10.1186/s40851-026-00267-6)
Supplement: Supplementary file 8 — Supplementary Material 8. Supplementary Table S3. PCR primers used for quantitative RT-PCR [file 40851_2026_267_MOESM8_ESM.pdf]

Supplementary Table S3. PCR primers used for quantitative RT-PCR.

Genes	Forward	Reverse
Gb'per	5'-AAGCAAGCAAGCATCCTCAT-3'	5'-CTGAGAAAGGAGGCCACAAG-3'
Gb'tim	5'-TCTCTCCAATGCTGTGTGAT-3'	5'-CTGGATCAGGAACTTTAGCACTTT-3'
Gb'cry2	5'-AGCACCATCACACACTTCACA -3'	5'-CACTCAGCGCAATCCACAC-3'
Gb'rpl18a	5'-GCTCCGGATTACATCGTTGC-3'	5'-GCCAAATGCCGAAGTTCTTG-3'
