# Supplementary material for: Knockout analysis of period and timeless and EGFP-based visualization of per-expressing clock cells in the cricket circadian clock
Source: Zoological Lett. 2026 Jul 7;12:12. doi: 10.1186/s40851-026-00267-6 (PMC13360532; doi:10.1186/s40851-026-00267-6)
Supplement: Supplementary file 9 — Supplementary Material 9. Supplementary Table 4. Numbers of per-egfp-expressing cells in the optic lobe [file 40851_2026_267_MOESM9_ESM.pdf]

Supplementary Table S4. Numbers of *per-egfp*-expressing cells in the optic lobe

	LaNdP	LaNd	LaNvP	LaNv	AMeNP	AMeN
Cell count (mean ± SD)	9.36 ± 0.92	3.63 ± 1.51	10.13 ± 1.56	3.63 ±1.69	7.5 ±0.93	4.63 ±1.06

*Data are presented as mean ± standard deviation (SD).

PDF-positive dorsal La neurons (LaNdP)

PDF-negative dorsal La neurons (LaNd)

PDF-positive ventral La neurons (LaNvP)

PDF-negative ventral La neurons (LaNv)

PDF-positive accessory medulla (AMe) neurons (AMeNP)

PDF-negative accessory medulla (AMe) neurons (AMeN)
